# Predictive equations for resting metabolic rate are not appropriate to use in Brazilian male adolescent football athletes

**DOI:** 10.1371/journal.pone.0244970

**Published:** 2021-01-14

**Authors:** Taillan M. Oliveira, Paula A. Penna-Franca, Christian H. Dias-Silva, Victor Z. Bittencourt, Fabio F. L. C. Cahuê, Sidnei J. Fonseca-Junior, Anna Paola T. R. Pierucci

**Affiliations:** 1 Graduate Program in Nutrition, Laboratory of Food Development for Special Health Purpose and Education (DAFEE), Nutrition Institute Josué de Castro (INJC), Federal University of Rio de Janeiro (UFRJ), Rio de Janeiro, Brazil; 2 Colégio de Aplicação, Universidade do Estado do Rio de Janeiro, Rio de Janeiro, Brazil; Instituto Politecnico de Viana do Castelo, PORTUGAL

## Abstract

High accuracy in estimating energy expenditure is essential for enhancing sports performance. The resting metabolic rate (RMR), as a primary component of total energy expenditure (TEE), is commonly estimated using predictive equations. However, these references may not be applicable to adolescent athletes. The purpose of this cross-sectional study was to analyse the differences between predicted RMR in relation to energy expenditure measured by indirect calorimetry (IC) among 45 Brazilian male adolescent football athletes. Indirect calorimetry (IC) and anthropometric (bioimpedance) measurements were recorded at a single visit to the laboratory after fasting overnight. The mean age was 15.6 ± 1.14 years, body mass was 63.05 ± 7.8 kg, and height was 172 ± 7.5 cm. The RMR values predicted by equations proposed by the Food and Agriculture Organization (FAO) (United Nations), Henry and Rees (HR), Harris Benedict (HB), and Cunningham (CUN) were compared with IC RMR values, by correlation analysis. The FAO and HR predictive equations yielded different values from IC (IC: 1716.26 ± 202.58, HR: 1864.87 ± 147.78, FAO: 1854.28 ± 130.19, p = 0.001). A moderate correlation of 0.504 was found between the results of HB and IC. In the survival-agreement model, the CUN equation showed low disagreement with the IC RMR, with error values between 200 and 300 kcal/day. The results showed that HB and CUN yielded similar values as IC, with the CUN equation showing low disagreement with IC; hence, adolescent athletes should undergo evaluation with precise laboratory methods to ensure that accurate information about RMR is recorded.

## 1. Introduction

To ensure optimal control of body composition and maximal sports performance, it is essential to balance energy intake with energy expenditure. Estimating the total daily energy expenditure (TEE) can be challenging for nutritionists because athletes’ nutritional goals and requirements are not static over the training year [1].

Daily TEE is composed of basal or resting metabolic rate (BMR or RMR), which refers to the energy cost of essential life processes (60–75% of the TEE), diet-induced thermogenesis (the energy expended to digest, absorb, and convert food, ~10%), and the energy expended during physical activities (activity energy expenditure, ~15–30%) [2, 3]. Although there are objective methods for measuring the TEE, such as doubly labeled water and indirect calorimetry (IC), these can be impracticable because of the high cost of the equipment and related maintenance. Thus, in practical field approaches, the TEE is generally estimated subjectively using predictive RMR equations.

Some of the equations proposed in the literature [4–7] are commonly used to estimate athletes’ RMR. These equations were mainly developed in studies carried out in North America [4, 5], while in some studies, the nationality of the participants has not been stated [6, 7]. Moreover, all of the studies included children, adults, and older populations and did not analyse adolescents exclusively [4–7]. As a result, the estimations of athletes’ RMR produced by these equations have questionable reliability. For several adolescent athletes, estimating the RMR is important to determine weight and height gain as well as for maintaining health and suitable body composition for the said modality [1].

Body composition is an important parameter for football players, who start their competitive careers in adolescence, and from that early age, they are subjected to constant and prolonged physical exhaustion. Football is an endurance sport [8], characterized by intermittent activity with bursts of intense effort [9]. The players’ activities during a match include standing, walking, jogging, cruising, sprinting, and backing, and the time spent on each of those activities depends on the player’s field position [10]. Athletes may compete two or three times a week, and the number of games played impacts their response to training [11], including energy demands. Thus, it is important to estimate the RMR correctly because it is the main variable for estimating the TEE.

In current clinical practice, predictive equations are widely used for estimating the RMR of adolescent athletes [1]. Previous studies have shown that RMR equations may not produce accurate results for this population, and there is no agreement between studies on the most suitable equation [12]. To date, no study has evaluated the accuracy of predictive equations in estimating the RMR in Brazilian adolescent football players. We hypothesized that predictive equations and IC, which is considered the gold standard for estimating the RMR, yield different RMR values [13]. Therefore, the aim of this study was to investigate the suitability of four predictive equations for evaluating the RMR of adolescent football players in Brazil using IC as the standard method.

## 2. Methods

In all, 45 male players from the under-15 and under-17 categories of a first division team in the Rio de Janeiro State Association Football Championship participated in this cross-sectional study. The inclusion criteria were training load of at least 4 hours for 5 days each per week, engaged in practice for at least 1 year prior to recruitment, and competed in at least one official national championship season. After club approval and meeting with the coaching committee, a meeting was held with the athletes for recruitment. The participants and their parents or legal guardians were informed about the experimental procedures and possible risks associated with the study, and informed consent was obtained in writing. The participants completed the testing procedures on a single day. Four athletes were evaluated per day for 3 weeks at the Nutrition Evaluation Laboratory of the Federal University of Rio de Janeiro. The football players underwent anthropometric, body composition, and indirect calorimetry measurements after fasting for 8 hours and 24 hours of rest from training. The study was approved by the Research Ethics Committee of the Clementino Fraga Filho University Hospital, Rio de Janeiro Federal University, Brazil (CAAE 58179716.3.0000.5257).

### Anthropometric and body composition measures

Body mass was assessed using a digital scale platform without an anthropometric ruler, Filizola® brand, accurate to 0.05 kg and 150 kg. Height was measured using an Alturexata® portable stadiometer, with a bilateral scale in millimeters (resolution of 1 mm), and field of use of 0.35 to 2.13 m. The participants were asked to remain barefoot for the measurements [14]. The body mass index (BMI) was calculated. Body fat percentage, body mass, fat-free mass (FFM), and fat mass (FM) were measured by bioimpedance (Byodinamics® 450) [15]. The participants were instructed to avoid consuming alcohol or caffeine on the day prior to the test, to not engage in exercise, to observe 8 hours of fasting, and avoid using diuretic medications. The participants were asked to lie down in a relaxed manner, in the supine position, barefoot without any metal adornment, with the hands leaning away from the body, and legs hip-distance apart.

### Indirect calorimetry measures

IC was carried out as per Compher’s [16] protocol. The participants were instructed to avoid consuming any thermogenic food, supplements, stimulants, sleep or appetite inhibitors, analgesics, or other substances known to affect RMR 1 day before the test. In addition, they were instructed not to take part in any physical practice on the day prior to the test and fast for 8 h before giving the measurements. On arrival to the laboratory, the participants were interviewed individually by a specialised nutritionist, and adherence to the RMR measurement protocol was confirmed. For IC, the Vmax Encore 29 System (VIASYS Healthcare Inc., Yorba Linda, CA) calorimeter was used. The measurements were taken in the morning, with the athletes in the supine position. A maximum of four participants were examined per day. Oxygen consumption (VO_2_) and carbon dioxide production (VCO_2_) values were collected by canopy and checked continuously for 30 min. For calculation purposes, the first 10 minutes were discarded to ensure data homogeneity. The VO_2_ and VCO_2_ values were used in the equation proposed by Weir [17] [Eq 1].

Equations:

$$RMR\;kcal/day=(3.9\;x\;VO_{2}(ml/\min)+1.1\;x\;VCO_{2}(ml/\min))x1440$$ (Eq 1)

### Predictive RMR equations

The following predictive equations were used:

Harris&Benedic(1919):66.4730+(13.7516xW)+(5.0033xH)−(6.7550xA) (Eq 2)

Cunningham(1980):581.6+21.6xFFM (Eq 3)

Henry&Rees(1991):(0.084xW+2.122)x239 (Eq 4)

FAO(2004):17.686xW+658.2 (Eq 5)

Legend: weight (W), height (H), age (A), fat-free mass (FFM)

### Statistical analyses

The Statistical Program for Social Sciences, version 20.0 (SPSS, Chicago, IL, USA) was used for statistical analyses. The methods were compared by difference analysis, correlation, degree of agreement, and survival-agreement. Additionally, IC data were also compared against age, anthropometric, and body composition variables to determine associations among these parameters. The Kolmogorov-Smirnov test was used to establish data normality, and the results were expressed as mean and standard deviation. Repeated measures one-way ANOVA was applied to compare mean RMR values between predictive equations and IC at 95% significance (p < 0.05). Pearson’s correlation was used to compare the values from predictive equations; IC values, and the variables of weight, height, age, BMI, FM, and FFM. The coefficients of correlation were considered strong (r > 0.8), moderate (r < 0.8 and >0.5), or weak (r < 0.5). Additionally, the Bland–Altman plot [18] and survival agreement [19] were used to ascertain the degree of agreement between the aforementioned methods.

## 3. Results

**Fig 1:**
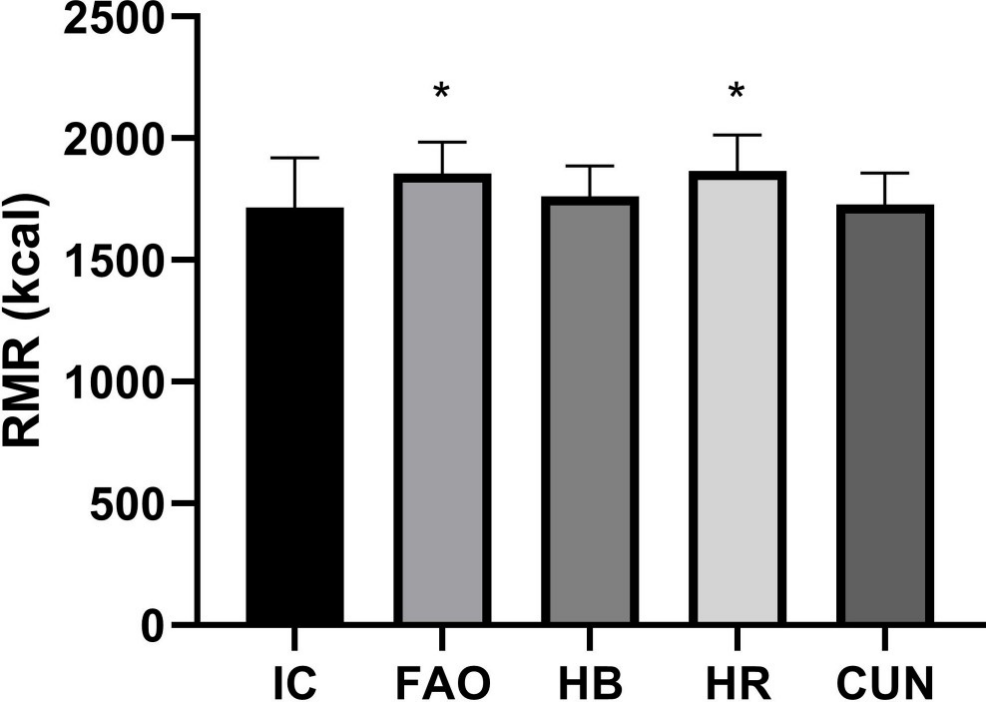
Comparison between predictive equations and indirect calorimetry. IC—Indirect calorimetry, HB—Harris & Benedict, C—Cunningham, HR—Henry & Rees, FAO—Food and Agriculture Organization (FAO). * p < 0.05 comparing to IC using 2-tails Student`s *t* test.

**Fig 2:**
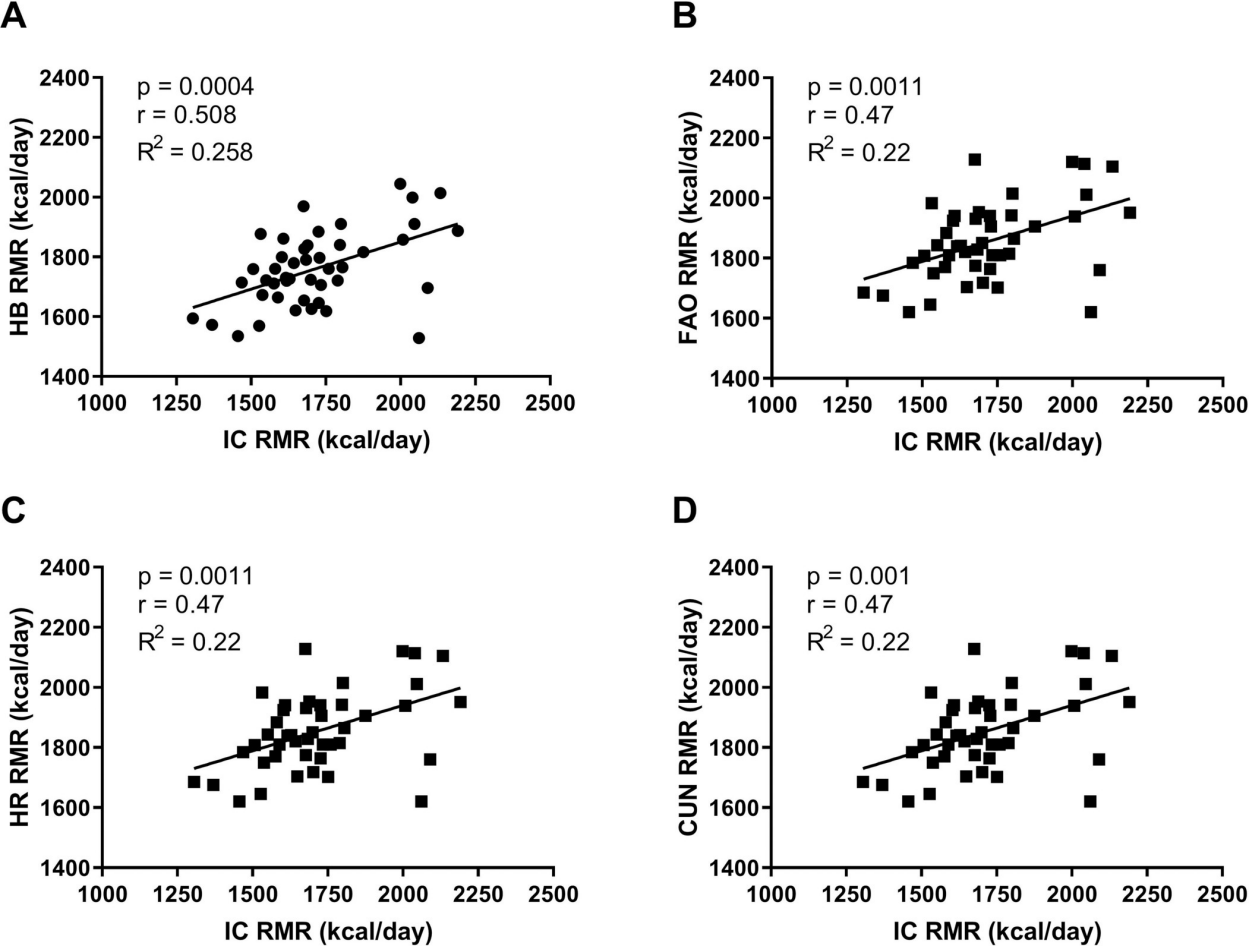
Correlation of predictive equations and indirect calorimetry. Panel A—Harris & Benedict (HB), Panel B—Food and Agriculture Organization (FAO), Panel C—Henry & Rees (HR), Panel D—Cunningham (C).

The characteristics of the participants are shown in Table 1. The RMR predicted by the HB and CUN equations was not different from the IC RMR values, but values obtained from the HR and FAO equations were statistically different (IC × FAO p = 0.0002, IC × HR p = 0.0001) from IC RMR values (Fig 1). Moderate correlations were found between the RMR values from the equations and IC, with a high correlation to the values from the HB equation (Fig 2).

**Table 1:** Age and body characteristics of male adolescent soccer players (n = 45).

Variables	Mean ± SD
Age (years)	15.69±1.41
Height (cm)	173.00±7.53
Body mass (kg)	67.63±7.36
BMI (kg/m^2^)	22.32±1.61
Fat mass (kg)	21.35±4.98
Fat mass (%)	14.53±4.06
Fat-free mass (kg)	53.10±5.95
Fat-free mass (%)	78.26±4.99

**Fig 3:**
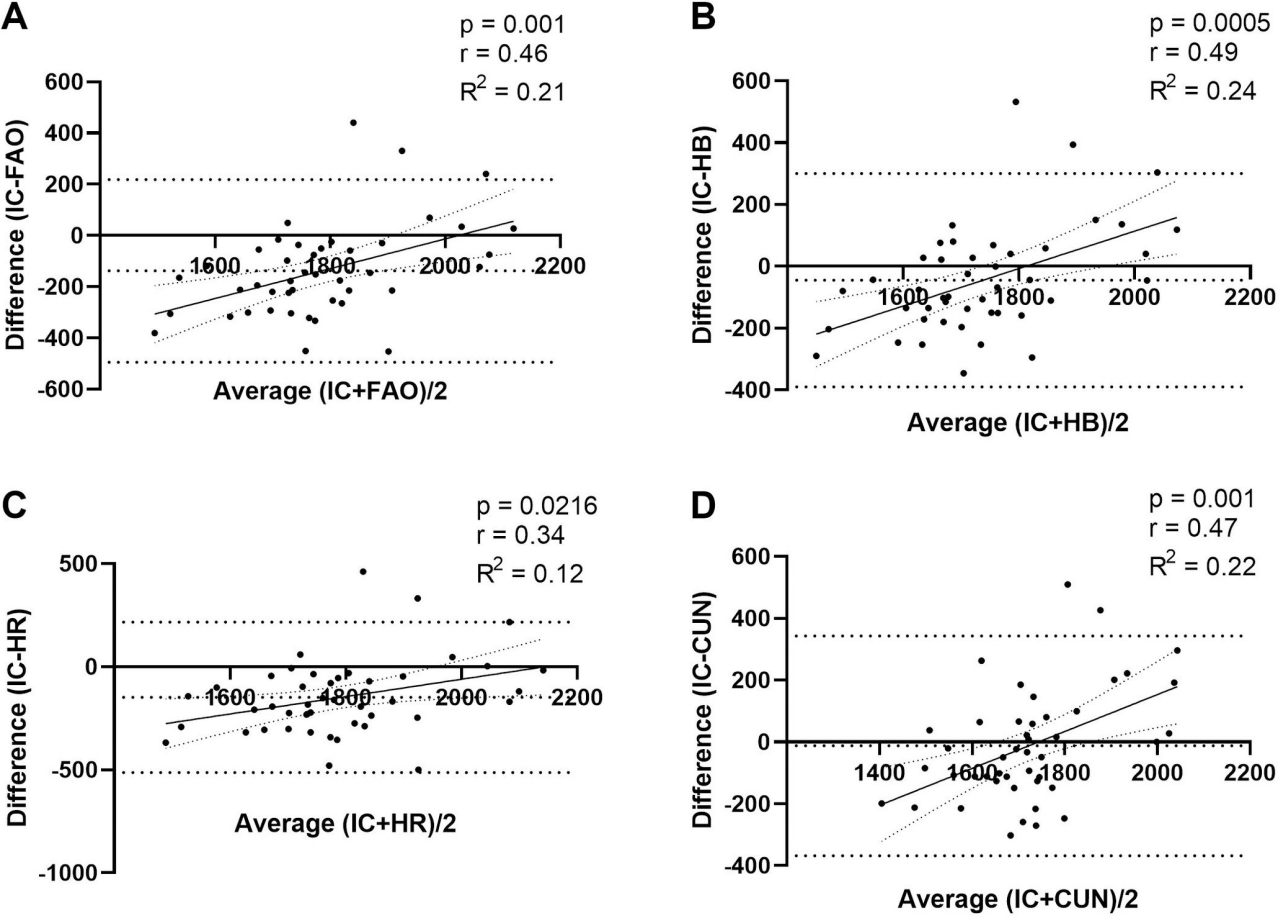
Bland–Altman scatter plots of measured RMR (IC) and predictive RMR equations. Panel A—Food and Agriculture Organization (FAO), Panel B—Harris & Benedict (HB), Panel C—Henry & Rees (HR), Panel D—Cunningham (CUN). Horizontal dotted line denotes the mean difference between measured RMR and respective predictive equation; and spaced dotted horizontal lines denote the 95% CI of the limits of agreement.

The Bland–Altman scatter plots in Fig 3, which compare data at an individual level, showed a significant difference between IC RMR and predicted RMR values. The differences and confidence intervals (95% CI) between the predicted values and IC values were, respectively, 138.02 ± 182.07 and 84.82–191.22 for FAO, 44.41 ± 176.11 and -7.04–95.87 for HB, 148.61 ± 186.24 and 94.20–203.02 for HR, and 12.25 ± 181.29 and -40.72–65.22 for CUN. The values obtained from CUN and HB were closer to the IC RMR values than those obtained from the other equations.

**Fig 4:**
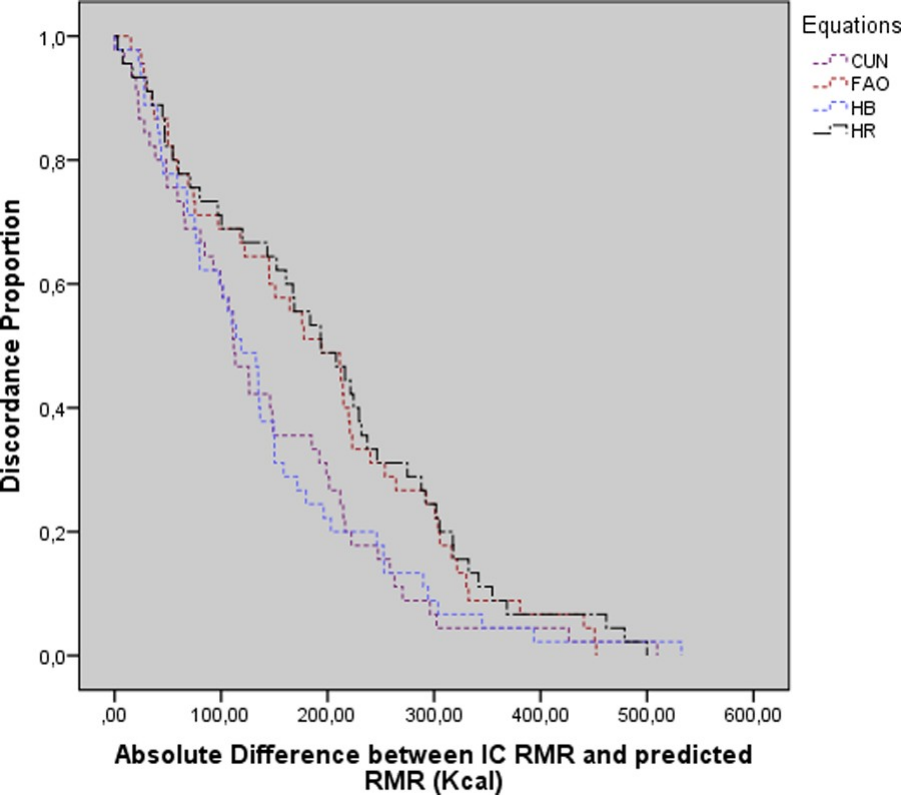
Survival-agreement plot for indirect calorimetry and predictive equations. Cunningham (CUN), Food and Agriculture Organization (FAO), Harris Benedict (HB), Henry and Rees (HR).

The agreement-survival results plotted in Fig 4 show a lack of agreement between the RMR values obtained from the equations and IC, with a variation of 200 to 400 kcal between them. On comparing the RMR values from IC and the equations, the CUN equation showed lower variation than the others, of 200 to 300 kcal/day, while for the other equations, the discrepancy was 300 to 400 kcal/day, at a disagreement level of 0.2 (20%) [19] (Fig 4).

The Pearson correlations between the body composition variables and IC values were determined. Except for height (r = 0.493) and body mass (r = 0.446), all variables showed moderate correlations to IC values (age: r = 0.282; BMI: r = 0.130; FM: r = 0.396; FFM: r = 0.396).

## 4. Discussion

We aimed to comparatively analyse the suitability of RMR prediction equations and RMR measured by IC in adolescent male football athletes. Our main finding was that the equations yielded RMR values with variations of 200 to 400 kcal from the IC RMR values. The HB and CUN equations showed moderate agreement compared to the FAO and HR equations. These results are in line with previous findings about differences between values obtained from IC and prediction equations [20–22].

The RMR is the main variable used to calculate daily TEE, so accurate calculation is a crucial requirement [1]. The equations investigated in this study yielded similar coefficients of variation (7.02–7.92%), which were lower than that yielded by IC (11.80%). This indicates that the equations have minimal capacity of detecting differences among athletes and each athlete’s actual energy individualised needs as compared to IC. Furthermore, sports training demands different levels of physical exertion and predicted energy expenditure [23]. In football, a variation of 1000–2000 kcal [24] in energy expenditure may be expected depending on the player’s position during a game. Individualised nutritional counselling is an essential tool in training programs for high-performance football players. Differences of 200–400 kcal in the predicted RMR can lead to weight gain or loss and to changes in body composition [1]. Besides, it can impact an athlete’s daily energy availability [20] and be detrimental to his/her sports performance, recovery, and overall health [25–27].

Although the HB and CUN equations returned better values, the results cannot be considered accurate. Studies conducted with athletes of different modalities have shown that the RMR prediction equations proposed by FAO and HR did not agree with IC RMR values [12, 23]. Loureiro et al. [27] investigated this in modern pentathlon athletes using the same statistical procedures as used in the current study and found similar results as the current study. Cherian et al. [12] also found the CUN and HB equations to be the best for predicting the RMR in a small sample of adolescent Indian football players. It is also important to note the differences between the characteristics of the study sample in the present study and of the populations on which the equation studies were based on [12, 23, 27].

Bland–Altman plots were used to present the degree of association between the values obtained from IC and the predictive equations [17], while survival-agreement plots were used to quantify the average overestimation or underestimation margin of each predictive equation [18]. The Bland–Altman plots showed that the equations did not agree with IC, with HB showing the best graphical association. However, studies using the same graphical demonstrations found acceptable results with the CUN equation, contrary to our data [12, 23, 27–29]. The survival-agreement plots showed that the accepted discordance proportion of 0.2 (20%) [19] induced an underestimation of 200 kcal in RMR estimation for both the HB and CUN equations.

Correlations between IC and anthropometric parameters were weak or moderate. Sagayama et al. [30] examined whether body composition estimated by bioimpedance influenced the RMR values; they found that weight and body mass were not significantly associated with RMR data, similar to our study. In general, the use of predictive equations to assess athletes’ RMR is criticized because their body composition interferes with the results since the equations were proposed for non-athlete populations [31].

Our study evaluated whether the equations presented differences between their means in a way that the variation could impair the assessment of a group of athletes. It also verified the agreement between the methods at both the individual and group levels. Finally, we evaluated the disagreement between the methods to verify the margin of error that a practitioner in clinical practice would have to consider when choosing any of the methods.

### Limitations

Evaluating FM in adolescent athletes is a challenge in nutritional monitoring [32]. Studies aimed at determining the most accurate methods for assessing body composition in athletes are still ongoing and there is a gap in the knowledge [33, 34], moreover, the four-compartment model is too complex to be used in day-to-day sports activities [35, 36].

Another possible limitation of this study is that nutrient intake was not assessed. Although the athletes were interviewed on food and supplement consumption to ensure RMR protocol compliance, the data were not analysed as nutrient intake. According to Madzima et al. [37], protein and carbohydrate ingestion prior to analysis by IC can influence RMR measurements.

## 5. Conclusion

In summary, this study evaluated differences between the predicted and IC RMR values. The present results conform with previous findings in the literature on athletes and diverse sports modalities, showing that the use of equations to predict RMR has practical limitations. The predictive equations tested in this study showed differences of 200–400 kcal from the IC RMR values. Therefore, when using predictive equations, the athletes’ energy balance as well as maintenance of body composition, physical performance, and recovery may be compromised. Athletes should undergo RMR evaluation with precise laboratory methods to obtain appropriate dietary prescriptions.

### Novelty statement

The energy needs of football adolescent athletes can be incorrectly estimated using RMR equations. The difference between the RMR values from IC and predictive equations was up to 400 kcal.

### Practical applications

When choosing a predictive equation to determine an athlete’s RMR, the nutritionist must consider its margin of error and field applicability.

## Supporting information

S1 File (DOCX)
