# Supplementary material for: Predictive equations for resting metabolic rate are not appropriate to use in Brazilian male adolescent football athletes
Source: PLoS One. 2021 Jan 14;16(1):e0244970. doi: 10.1371/journal.pone.0244970 (PMC7808585; doi:10.1371/journal.pone.0244970)
Supplement: S1 File — (DOCX) [file pone.0244970.s001.docx]

|  | IC | FAO | HB | HR | C |
| --- | --- | --- | --- | --- | --- |
| 1 | 2061,037 | 1620,318 | 1528,724 | 1599,292 | 1551,44 |
| 2 | 1628,817 | 1841,393 | 1727,133 | 1850,242 | 1845,2 |
| 3 | 1369,695 | 1675,145 | 1572,853 | 1661,528 | 1581,68 |
| 4 | 1797,085 | 1942,204 | 1840,791 | 1964,676 | 1611,92 |
| 5 | 1589,644 | 1809,559 | 1664,858 | 1814,106 | 1715,6 |
| 6 | 1759,527 | 1809,559 | 1760,168 | 1814,106 | 1700,48 |
| 7 | 1677,733 | 1931,592 | 1827,284 | 1952,63 | 1804,16 |
| 8 | 2046,024 | 2011,179 | 1910,181 | 2042,972 | 1823,6 |
| 9 | 1642,5 | 1820,17 | 1779,174 | 1826,151 | 1691,84 |
| 10 | 1688,633 | 1952,815 | 1838,783 | 1976,721 | 1802 |
| 11 | 1684,035 | 1829,013 | 1790,303 | 1836,189 | 1706,96 |
| 12 | 1468,407 | 1784,798 | 1714,649 | 1785,999 | 1683,2 |
| 13 | 1726,654 | 1763,575 | 1646,614 | 1761,908 | 1717,76 |
| 14 | 1702,224 | 1717,591 | 1625,869 | 1709,71 | 1735,04 |
| 15 | 1675,612 | 2127,907 | 1969,921 | 2175,474 | 1922,96 |
| 16 | 2090,34 | 1760,038 | 1696,648 | 1757,893 | 1663,76 |
| 17 | 2039,46 | 2113,758 | 1998,944 | 2159,413 | 2011,52 |
| 18 | 1616,555 | 1839,625 | 1730,761 | 1848,235 | 1765,28 |
| 19 | 1305,081 | 1685,757 | 1594,865 | 1673,574 | 1503,92 |
| 20 | 1537,902 | 1749,426 | 1672,639 | 1745,847 | 1557,92 |
| 21 | 1805,436 | 1864,385 | 1765,275 | 1876,341 | 1659,44 |
| 22 | 1455,945 | 1620,318 | 1535,479 | 1599,292 | 1540,64 |
| 23 | 1602,875 | 1924,518 | 1799,27 | 1944,6 | 1873,28 |
| 24 | 1677,024 | 1774,187 | 1654,865 | 1773,954 | 1769,6 |
| 25 | 1550,394 | 1843,162 | 1722,006 | 1852,25 | 1661,6 |
| 26 | 1648,701 | 1703,443 | 1620,873 | 1693,65 | 1583,84 |
| 27 | 1875,478 | 1905,063 | 1816,663 | 1922,516 | 1776,08 |
| 28 | 1729,08 | 1905,063 | 1796,904 | 1922,516 | 1706,96 |
| 29 | 1607,948 | 1940,435 | 1860,927 | 1962,668 | 1709,12 |
| 30 | 1998,662 | 2120,832 | 2044,468 | 2167,443 | 1998,56 |
| 31 | 2132,577 | 2104,915 | 2013,832 | 2149,375 | 1940,24 |
| 32 | 1532,033 | 1982,881 | 1877,17 | 2010,85 | 1834,4 |
| 33 | 1699,217 | 1850,236 | 1724,003 | 1860,28 | 1847,36 |
| 34 | 1576,188 | 1770,649 | 1711,154 | 1769,938 | 1687,52 |
| 35 | 1618,765 | 1839,625 | 1720,755 | 1848,235 | 1730,72 |
| 36 | 1725,471 | 1940,435 | 1884,19 | 1962,668 | 1773,92 |
| 37 | 1789,903 | 1814,864 | 1721,515 | 1820,128 | 1773,92 |
| 38 | 2007,853 | 1938,666 | 1857,8 | 1960,66 | 1806,32 |
| 39 | 1506,877 | 1807,79 | 1759,542 | 1812,098 | 1614,08 |
| 40 | 2191,11 | 1951,047 | 1887,438 | 1974,714 | 1894,88 |
| 41 | 1580,267 | 1883,84 | 1760,137 | 1898,425 | 1838,72 |
| 42 | 1751,571 | 1701,674 | 1618,748 | 1691,642 | 1488,8 |
| 43 | 1800,423 | 2014,716 | 1910,43 | 2046,987 | 1719,92 |
| 44 | 1734,129 | 1809,559 | 1706,135 | 1814,106 | 1668,08 |
| 45 | 1527,057 | 1645,079 | 1569,74 | 1627,399 | 1488,8 |

**SUPLEMENTARY DATA**

**Table 1 Participant´s measured and predicted rest metabolic rate (RMR) values.**

Indirect Calorimetry (IC), Food and Agriculture Organization (FAO), Harris Benedict (HB), Henry and Rees (HR), Cunnigham (C).


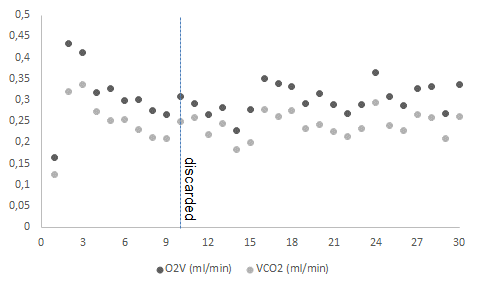


**Suplementary Figure O_2_V and VCO_2_ results by indirect calorimetry of one of the participants.** The graph above represents the indirect calorimetry procedure. Oxygen consumption (O_2V_) and carbon dioxide production (VCO_2_) were collected by canopy and checked continuously for 30 minutes. To guarantee greater data homogeneity dots of the first ten minutes were discarded (represent o line).
